# Immune Checkpoint Inhibitors in Relapsed/Refractory Anaplastic Large Cell Lymphoma (ALCL): A Case Report and Review of the Literature

**DOI:** 10.1002/cnr2.70684

**Published:** 2026-09-06

**Authors:** Constantin Klenner, Josef Singer

**Affiliations:** ^1^ Karl Landsteiner University of Health Sciences Krems Austria; ^2^ Department of Internal Medicine II, University Hospital Krems Karl Landsteiner University of Health Sciences Krems Austria

## Abstract

**Background:**

While immune checkpoint inhibitors (ICI) have transformed the landscape of clinical oncology, their application in rare malignancies such as anaplastic large cell lymphoma (ALCL) remains limited.

**Case:**

We report a patient with relapsed/refractory (r/r) ALCL treated at the University Hospital Krems who achieved sustained complete remission under PD‐1 blockade after multiple prior lines of therapy. In addition, we performed a literature review and identified three previously published cases. Across the four reported patients, all achieved complete remission and remained progression‐free throughout their respective observation periods, ranging from 4 to 33 months. Where patient‐level safety data were available, reported adverse events were manageable and consistent with known immune‐related toxicities.

**Conclusion:**

Although based on very limited data, these observations suggest potential clinical activity of PD‐1/PD‐L1 inhibition in r/r ALCL. They also underscore the urgent need for prospective studies to validate efficacy and safety in larger cohorts. Future research should incorporate biomarker analyses to refine patient selection and explore rational treatment combinations, such as ICI with Brentuximab Vedotin, in order to broaden therapeutic opportunities for this challenging disease.

## 1 Background

Immune checkpoint inhibitors (ICI) have significantly advanced clinical oncology, yet their application in rare entities remains scarcely explored. One such entity is anaplastic large cell lymphoma (ALCL), classified as ALK+/ALK−, breast implant‐associated (BIA‐ALCL), or primary cutaneous (pcALCL). For systemic ALCL, first‐line treatment generally consists of anthracycline‐based chemotherapy, with BV‐CHP (Brentuximab Vedotin, Cyclophosphamide/Hydroxydaunorubicin/Prednisone) representing the preferred regimen for CD30‐positive disease in adult patients [1]. In relapsed/refractory (r/r) disease, options include BV, autologous/allogeneic SCT, ALK inhibitors for relapsed ALK+ disease [2], or individualized approaches [1]. BV achieves complete remission (CR) in ~60% of r/r ALCL [3]. However, if CR is not achieved, median overall survival is only 9.5 months [4], underscoring the need for alternative strategies.

When standard treatment options have been exhausted, comprehensive genomic profiling may identify biomarkers that provide a biological rationale for individualized therapeutic approaches, including biomarkers potentially associated with response to ICI, such as PD‐L1 expression, microsatellite instability (MSI), or high tumor mutational burden (TMB). To our knowledge, this is the first reported case of r/r ALCL in which treatment selection for PD‐1 blockade was guided by genomic profiling demonstrating high TMB. We additionally provide a review of the literature, identifying only three previously published adult cases treated with ICI.

Detailed methods of the literature search and results are described in the Supporting Information. Table S1 shows the eligibility criteria for the literature review. Table S2 represents the initial PubMed search. The relevant information of each included study is presented in Table S3. Written informed consent from the patient was obtained for the publication of case details and use of images.

## 2 Case Presentation

A 75‐year‐old woman first presented in May 2017 at the University Hospital Krems with chronic diarrhea and weight loss. Initial workup for infections, celiac disease, inflammatory bowel disease, and colonoscopy was negative.

Despite an unrevealing diagnostic work‐up, symptoms worsened and in July 2017 she developed a small‐bowel obstruction requiring surgical resection. Histopathology revealed ALCL, ALK−, Stage IVB. First‐line CHOP (Cyclophosphamide, Hydroxydaunorubicin, Vincristine, Prednisone; 8 cycles) achieved CR, but relapse occurred 3 months after therapy completion (February 2018). Second‐line BV (1.8 mg/kg body weight, no dose reductions) led to CR after eight cycles (August 2018). Six months after stopping BV (February 2019), the disease relapsed again, prompting re‐initiation of BV for an additional 33 cycles (1.8 mg/kg body weight, no dose reductions). The patient once again achieved a complete metabolic response (CMR), and treatment was discontinued in February 2021.

Fifteen months later, in May 2022, a third relapse occurred. Due to peripheral neuropathy (Grade 1–2), BV was withheld. An individualized treatment plan using Lenalidomide was chosen (15 mg/day for 21 days of a 28‐day cycle), resulting in temporary disease stabilization. The patient completed seven cycles before disease progression occurred in November 2022.

BV was attempted once more (1.8 mg/kg body weight, six cycles from April to August 2023, without dose reductions), but this time failed to elicit a response. At this point, comprehensive genomic profiling of circulating tumor DNA (ctDNA) using FoundationOne Liquid was performed in September 2023 and revealed a high TMB of 23 mutations/megabase. Given the high TMB and limited remaining treatment options, Nivolumab (240 mg every 2 weeks, later modified to 480 mg every 4 weeks) was started. Clinical improvement was observed within 2 months. At the first response assessment in March 2024, approximately 6 months after nivolumab initiation, she achieved CMR.

In April 2024, approximately 6 months after nivolumab initiation, the patient developed Grade 2 immune‐related thyroiditis, which was managed with thyroid hormone replacement therapy. Importantly, this immune‐related adverse event did not necessitate discontinuation of Nivolumab.

In December 2025, the patient suffered a myocardial infarction requiring percutaneous coronary intervention with stent implantation. By that time, she had already completed 25 months of Nivolumab therapy, with the last dose administered in November 2025. Given the sustained CMR, no further nivolumab was administered, and the patient transitioned to follow‐up care.

At the most recent follow‐up (July 2026), 8 months after the end of ICI therapy, the patient remains in CMR; repeat liquid biopsy showed no detectable tumor‐associated alterations (FoundationOne Liquid), and she is in good overall condition (ECOG 1).

A detailed timeline (month/year) summarizing diagnosis, relapses, treatment changes, and responses can be found in Table 1.

**TABLE 1:** Timeline summarizing diagnosis, relapses, treatment changes, and responses.

Month/year: Diagnoses, relapses	Therapies (Starting date–End date) >> responses
July 2017: Initial diagnosis, 75a ALCL ALK–, Stage IVB	First‐Line treatment with CHOP, 8 cycles, 08/2017–12/2017 >> complete response (CT scans)
02/2018: First relapse	Leading to Second‐Line treatment with BV, 8 cycles, 03/2018–08/2018 >> complete response (CT scans)
02/2019: Second relapse	Re‐initiation of BV, 33 cycles 04/2019–02/2021 >> complete metabolic remission (PET/CTs)
05/2022: Third relapse	Treatment with Lenalidomide, 7 cycles, 05/2022–11/2022 >> stable disease (CT scans)
11/2022: Disease progression on lenalidomide	Re‐Challenge of BV, 6 cycles, 04/2023–08/2023 >> progressive disease (CT scans)
08/2023: Disease progression on BV	Treatment with Nivolumab, 10/2023–11/2025 >> complete metabolic remission (PE/CT 03/2024, confirmed in serial CT scans as well as PET/CT 11/2025) The last dose of nivolumab was administered in November 2025. In December 2025, the patient suffered a myocardial infarction requiring percutaneous coronary intervention with stent implantation. Given that she had already completed 25 months of nivolumab treatment and remained in sustained complete metabolic remission, treatment was not resumed and the patient transitioned to follow‐up care.
Last follow‐up 07/2026	84a, no active antineoplastic treatment >> sustained complete metabolic remission, no detectable tumor‐associated alterations on repeat ctDNA analysis; ECOG 1

### 2.1 Cases Reported in the Literature

Chan et al. [5] report a 35‐year‐old woman with ALK− ALCL who failed ProMACE‐CytaBOM, then received Cisplatin/Ifosfamide/Gemcitabine/Etoposide/L‐Asparaginase and subsequently underwent autologous SCT. The disease relapsed after 11 months. Thus, BV was administered and CR was achieved after 5 cycles. Although she then received allogeneic SCT, relapse occurred 3 months later, leading to the decision to begin pembrolizumab. She achieved CR after ~70 days and remained in CR at the last reported follow‐up of approximately 4 months. The authors noted elevated transaminases, treated with methylprednisolone as the only side effect.

Hebart et al. [6] describe a 19‐year‐old man with ALK+ ALCL who relapsed despite extensive prior treatments, including cyclophosphamide/daunorubicin/vincristine/etoposide/prednisolone/BV/Crizotinib and autologous SCT. Four months after SCT, his condition worsened, prompting nivolumab initiation. The response was rapid and marked, with CR achieved within 1 month. He remained relapse‐free during the 9‐month follow‐up. The only reported side effect was pneumonitis after 12 cycles, treated with corticosteroids.

Barta et al. [7] included one ALK− ALCL patient in a phase‐2 trial investigating PD‐1/PD‐L1 inhibition in r/r T‐cell lymphomas. As this was a single patient within a larger study, limited patient‐level information is available. The patient's age, sex, and prior therapies were not reported separately. Although the trial was halted early due to limited efficacy (CRR 24%), a notable finding was that the ALCL patient remained in CR for 18 months at publication. It is not defined when CR was achieved; only the trial‐wide median of 114 days (range 82–146) is reported. Due to the study design, no statements about ICI safety in ALCL can be made, as side effects were not separated by disease.

## 3 Discussion

Our review identified only three previously published adult patients with r/r ALCL treated with ICI, highlighting the scarcity of available data. Given the poor prognosis of multiply relapsed ALCL, the durable responses described are noteworthy. All three previously published patients [5, 6, 7], as well as our patient, achieved CR and remained progression‐free throughout the reported observation periods of 4, 9, 18, and 33 months, respectively (Table 2).

**TABLE 2:** Overview of published adult cases of relapsed/refractory ALCL treated with ICI.

Source	Follow‐up after ICI initiation	PFS	Response	Side effects	CR after	Status at last follow‐up
Present Case	33 months	33 months	Complete Metabolic Remission	Grade 2 Thyroiditis	~6 months	Nivolumab discontinued after 25 months; sustained CMR 8 months after discontinuation
Chan et al. 2016 ALK− ALCL	4.1 months	4.1 months	Complete Response	Elevated transaminases	~2 months	On treatment
Hebart et al. 2016 ALK+ ALCL	9 months	9 months	Complete Response	Pneumonitis	> 1 month	On treatment
Barta et al. 2019 ALK− ALCL	18 months	18 months	Complete Response	Not separately reported for the ALCL patient	Not reported	On treatment

While not eligible to be included in the primary review of adult patients, Rigaud et al. [8] report the pediatric case of a 17‐year‐old male diagnosed with r/r ALK+ ALCL and treated with 3 mg/kg body weight of nivolumab every 2 weeks as third line therapy. The patient had a rapid and sustained clinical response. He no longer required oral analgesics within 10 days of the first dose and remained in CR 18 months after nivolumab initiation, at the time of publication.

These observations, although based on very limited data, suggest potential clinical activity of PD‐1 blockade in selected patients with r/r ALCL. Where patient‐level safety data were available, reported adverse events were manageable and consistent with the established immune‐related toxicity profile of PD‐1 inhibitors. Still, caution is required, particularly in patients treated after allogeneic SCT due to the increased risk of graft‐versus‐host disease.

A major limitation is the strong publication bias inherent to case reports. Unsuccessful treatments are considerably less likely to be published, precluding any conclusions regarding the true response rate or efficacy of PD‐1 blockade in ALCL. Therefore, our findings should be interpreted as hypothesis‐generating only.

To our knowledge, this is the first reported case of r/r ALCL in which treatment selection for PD‐1 blockade was guided by genomic profiling demonstrating high TMB. Biomarkers such as high TMB and MSI have emerged as predictive biomarkers for immune checkpoint inhibition in several solid tumors [9], although their role in ALCL remains unknown. In our case, high TMB provided the rationale for considering PD‐1 blockade in the context of limited therapeutic alternatives. There is currently no evidence supporting TMB as a validated predictive biomarker for immune checkpoint inhibition in ALCL or other T‐cell lymphomas. Therefore, treatment selection in our patient was extrapolated from tissue‐agnostic evidence supporting ICI use in selected TMB‐high advanced solid tumors [10] and should be regarded as hypothesis‐generating rather than evidence‐based.

Tumor‐cell PD‐L1 expression was not assessed at the time of treatment initiation because it is not an established predictive biomarker in ALCL. During preparation of this manuscript, retrospective immunohistochemical analysis of the original 2017 tumor sample demonstrated PD‐L1 expression in 95% of tumor cells. Whether this finding contributed to the favorable clinical response remains speculative.

Incorporating such biomarkers into clinical practice may help refine therapeutic decisions. Moreover, serial ctDNA assessment may warrant investigation as a tool for monitoring disease dynamics and treatment response in ALCL.

Finally, the biological heterogeneity of ALCL should be considered. In ALK‐positive ALCL, PD‐L1 expression is driven by oncogenic signaling downstream of ALK, with constitutive STAT3 activation playing a central role [11]. In contrast, ALK‐negative ALCL lacks this oncogenic driver, and PD‐L1 expression may be regulated through alternative mechanisms, including STAT3‐ and MYC‐dependent transcriptional programs [12]. This distinction highlights the importance of separate evaluation of ALK+ and ALK− subtypes in future studies.

## 4 Conclusion

This case, together with the limited published evidence, suggests that PD‐1/PD‐L1 blockade may provide clinically meaningful and durable disease control in selected patients with multiply relapsed or refractory ALCL after established treatment options have been exhausted. The sustained complete metabolic remission observed in our patient, including after discontinuation of nivolumab, highlights the potential relevance of immune checkpoint inhibition as an individualized salvage strategy in this otherwise difficult‐to‐treat setting.

However, the available evidence remains restricted to a small number of highly selected cases and is subject to substantial publication bias. ICI should therefore not yet be considered a standard treatment for r/r ALCL. In clinical practice, their use may be considered on an individual basis for patients without suitable established alternatives, preferably after multidisciplinary discussion and within a clinical trial or structured translational program. Biomarkers such as TMB and tumor‐cell PD‐L1 expression may provide a biological rationale for treatment selection, but their predictive value in ALCL remains unvalidated.

Prospective studies are needed to define the efficacy, safety, optimal treatment duration, and role of biomarker‐guided patient selection for PD‐1/PD‐L1 inhibitors in ALCL. Rational combination strategies, particularly with brentuximab vedotin, also warrant investigation because of their potentially complementary mechanisms of action.

## Author Contributions

**Constantin Klenner:** conceptualization, investigation, writing – original draft, methodology, validation, writing – review and editing, formal analysis, data curation. **Josef Singer:** conceptualization, investigation, writing – original draft, methodology, validation, writing – review and editing, formal analysis, data curation, supervision.

## Funding

The authors have nothing to report.

## Disclosure

The disclosed financial relationships had no involvement in the study design; collection, analysis, or interpretation of data; writing of the manuscript; or the decision to submit the manuscript for publication.

## Conflicts of Interest

J.S. has received honoraria from AbbVie, Amgen, Angelini, Gilead, Janssen, Kite, Merck, Merck Sharp & Dohme, Miltenyi, Novartis, Pfizer, Roche, and Servier for speaking engagements or consultancy. C.K. declares no conflicts of interest.

## Supporting information


**Table S1:** Extended PICO scheme.
**Table S2:** Search results dated 21.11.2024 via Pubmed.
**Table S3:** Extracted data items.

## Data Availability

Methods of the literature search and results are described in the Supporting Information. The relevant data supporting this article are presented within the article. The original data of the case report from our clinic cannot be publicly shared due to patient confidentiality, but can be made available by contacting the corresponding author, Josef Singer, upon reasonable request and subject to applicable legal and ethical restrictions.
