# Supplementary material for: Immune Checkpoint Inhibitors in Relapsed/Refractory Anaplastic Large Cell Lymphoma (ALCL): A Case Report and Review of the Literature
Source: Cancer Rep (Hoboken). 2026 Sep 6;9(9):e70684. doi: 10.1002/cnr2.70684 (PMC13547745; doi:10.1002/cnr2.70684)
Supplement: Supplementary file 1 [file CNR2-9-e70684-s001.docx]

***Supplementary Information***

***Methods***

Supplementary Table 1 shows eligibility criteria for the literature review, based on the extended PICO scheme ^1^. Information sources are PubMed and Cochrane. A case study is integrated into this review, since the patient meets the eligibility criteria.

*Supplementary Table 1: extended PICO scheme*

|  | **Inclusion** | **Exclusion** |
| --- | --- | --- |
| **Population:** | Adult Patients diagnosed with r/r ALCL | Pediatric patients |
| **Intervention(s):** | PD-1/PD-L1/ immunotherapy | --- |
| **Comparison(s):** | Brentuximab-Vedotin, BV-CHP | --- |
| **Outcomes:** | 5-year survival, 12-month survival, overall survival, progression free survival, complete remission rate | Non-clinical outcomes |
| **Setting:** | Clinical | Pre-/Postclinical |
| **Study design(s):** | Randomized controlled trials, Reviews, Retrospective Cohort Studies, Case studies | Cross-sectional Studies, Preclinical studies |
| **Publication date:** | All | None |
| **Language:** | English, German | All other |

Supplementary Table 2 represents the initial PubMed search without limitations for appropriate studies.

*Supplementary Table 2: Search results dated 21.11.2024 via PubMed*

| Search # | Query | Results | Time |
| --- | --- | --- | --- |
| 1 | lymphoma, large cell, anaplastic [MeSH Terms] AND refractory | 162 | 17:28:06 |
| 2 | lymphoma, large cell, anaplastic [MeSH Terms] AND recurrent | 330 | 17:28:09 |
| 3 | lymphoma, large cell, anaplastic [MeSH Terms] AND relapsed | 423 | 17:28:15 |
| 4 | lymphoma, large cell, anaplastic [MeSH Terms] AND (refractory/recurrent OR recurrent/refractory) | 2 | 17:28:50 |
| 5 | #1 OR #2 OR #3 OR #4 | 523 | 17:29:21 |
| 6 | Immune Checkpoint Inhibitors [MeSH Terms] | 12,820 | 17:29:36 |
| 7 | Programmed Cell Death 1 Receptor [MeSH Terms] | 13,330 | 17:29:46 |
| 8 | #6 OR #7 | 24,006 | 17:31:39 |
| 9 | #5 AND #8 | 1 | 17:35:51 |
| 10 | Microsatellite Instability [MeSH Terms] | 5,358 | 17:39:12 |
| 11 | tumor mutational burden [Title/Abstract] | 3,364 | 17:42:34 |
| 12 | #10 OR #11 | 8,468 | 17:43:56 |
| 13 | #5 AND #12 | 0 | 17:44:43 |

For data management all results were imported to EndNote® and processed from there. Titles and abstracts were examined if they fit specific, predefined inclusion and exclusion criteria (see Supplementary Table 1). Possibly relevant studies were read in full before making a final decision. To ensure that no relevant data was left out of a review, sources of included studies for suitable case reports were manually examined. These also have to fulfil the inclusion criteria.

Three published cases met the study criteria - two case reports and one patient from a phase 2 trial - alongside an additional case from our clinic.

The relevant information of each included study is presented in Supplementary Table 3.

*Supplementary Table 3: Extracted data items*

|  | Type of Study | Number of Participants | Primary Outcome | Secondary Outcome | Duration of Intervention |
| --- | --- | --- | --- | --- | --- |
| Barta (2019) ^2^ | Clinical Trial | n=18 | PFS | ORR, OS, Response Duration, Safety | Up to 18 months |
| Hebart (2016) ^3^ | Case Report | n=1 | / | / | 9 months |
| Chan (2016) ^4^ | Case Report | n=1 | / | / | 4,1 months |

If multiple similar studies were found, the clinical and procedural differences were evaluated before inclusion to ensure that they are suitable. Since this field is not explored as well as others yet, a narrative summary of results was deemed to be suitable with studies being evaluated individually.

***References***

1.	Eriksen MB, Frandsen TF. The impact of patient, intervention, comparison, outcome (PICO) as a search strategy tool on literature search quality: a systematic review. *J Med Libr Assoc*. 2018;106(4):420-431.

2.	Barta SK, Zain J, MacFarlane AWt, et al. Phase II Study of the PD-1 Inhibitor Pembrolizumab for the Treatment of Relapsed or Refractory Mature T-cell Lymphoma. *Clin Lymphoma Myeloma Leuk*. 2019;19(6):356-364.e353.

3.	Hebart H, Lang P, Woessmann W. Nivolumab for Refractory Anaplastic Large Cell Lymphoma: A Case Report. *Ann Intern Med*. 2016;165(8):607-608.

4.	Chan TS, Khong PL, Kwong YL. Pembrolizumab for relapsed anaplastic large cell lymphoma after allogeneic haematopoietic stem cell transplantation: efficacy and safety. *Ann Hematol*. 2016;95(11):1913-1915.
